# Flow and Mixing Behavior in a New Bottom Blown Copper Smelting Furnace

**DOI:** 10.3390/ijms20225757

**Published:** 2019-11-16

**Authors:** Pin Shao, Lepeng Jiang

**Affiliations:** School of Materials and Metallurgy, University of Science and Technology Liaoning, Anshan 114051, China; louwen.1984@163.com

**Keywords:** copper smelting, bottom blown, gas-liquid flow, mixing behavior, modeling

## Abstract

A mathematical model was developed to describe gas–liquid flow and mixing behavior in a new bottom blown oxygen copper smelting furnace, and the model validation was carried out through a water model experiment. The effects of different nozzle locations, nozzle numbers, and gas flow rates on the gas–liquid flow, gas total volume, and mixing efficiency were investigated. The results show that the gas–liquid two-phase flow and mixing time predicted by the present model agree well with the experimental data. When the nozzles are located near the center of the bath bottom, the gas total volume is larger, but the mixing efficiency is very low. With the increase of nozzle arrangement angle, the mixing time decreased. However, the excessive angle arrangement of nozzles exceeding 21° was found to be detrimental to the bubble residence time and mixing efficiency. With the increase in nozzle numbers from nine to 13, the gas total volume in the furnace increases, and the mixing efficiency does not change greatly. When the number of nozzles is further increased to 18, the mixing efficiency begins to decrease significantly. As the gas flow rate increases from 4.7 m^3^/h to 14.1 m^3^/h, the gas total volume in the furnace increases, and the mixing time is rapidly reduced from 314.5 s to 251.5 s. When the gas flow rate exceeds 18.8 m^3^/h, the gas total volume and mixing efficiency change little.

## 1. Introduction

**Figure 1:**
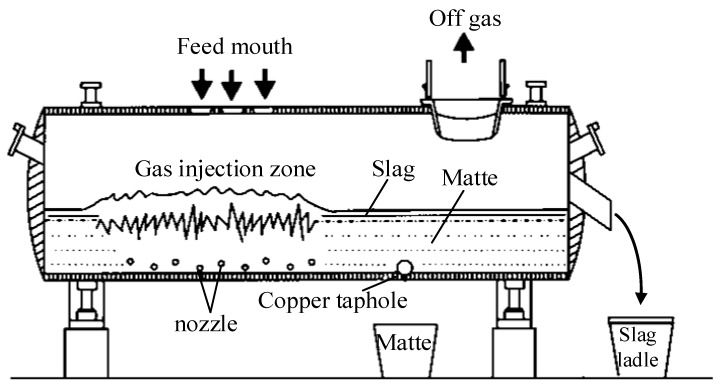
Schematic diagram of bottom blown copper smelting furnace.

Oxygen bottom blown copper smelting is a new technology where multiple oxygen nozzles are placed at the bottom of the furnace, and the oxygen-enriched air is directly injected into the blister copper layer for copper smelting to produce high-grade matte. As shown in Figure 1, in this process, the concentrate particles are added to the furnace from the feeding port, and then entangled and mixed by a high temperature flow into the interior of the bath. The smelting and slagging reactions of these particles with oxygen occur strongly in the injection zone. In recent years, this technology has been widely used in China because it has many advantages such as good kinetic conditions, low investment, high SO_2_ concentration for acid plants, strong adaptability of raw materials and low energy consumption [1,2,3,4,5]. However, as a new technology, the bottom blowing copper method also has some shortcomings. For example, the layout of the oxygen lance lacks theoretical basis, and the mixing and smelting efficiency in the molten pool need to be further improved. Furthermore, the intense splashing often occurs in molten pool which easily leads to blockage of the feed port. These problems are closely related to the phenomenon of fluid flow and mixing in the furnace, which needs to be studied deeply and systematically for efficient smelting.

At present, the gas-liquid flow in bottom blown copper furnace have been studied by scholars using numerical simulation [6,7,8,9] and water model experiments [10,11,12]. These studies provide a good theoretical basis for the design of the bottom blow nozzle. However, the understanding of the actual flow and mixing behavior in the furnace under different working conditions is still not comprehensive. For example, in the actual production process, 6 to 20 nozzles are always installed at the bottom of the furnace in one or two rows according to different smelting scales. In addition, the arrangement angle of the nozzle at the bottom of the furnace also changes from 0° to 28°, and the range of industrial gas flow rate is from 4000 to 12,000 Nm^3^/h. Shui [10] established a scaled-down 1:12 water model to describe the mixing phenomena in bottom blown copper smelting furnace. In this study, only the case of a single nozzle blowing was considered, and the impact of nozzle number, arrangement angle, and gas flow rate on flow and mixing efficiency in the furnace were not revealed and still need to be systematically studied.

The aim of present work is to develop a Euler–Euler mathematical model to describe the gas-liquid flow and mixing behavior in a bottom blown copper smelting furnace reasonably based on the verification of the water model. The impact of different nozzle arrangements, nozzle number, and gas flow rate on the gas-liquid flow and mixing behavior in a bottom blown copper smelting furnace are investigated, and proper operating factors are proposed.

## 2. Results and Discussion

In a bottom blown copper smelting furnace, the gas–liquid two-phase flow is the main reaction site of the molten pool, and the nozzle arrangement, nozzle number, and gas flow rate have a significance effect on the gas–liquid two-phase flow and mixing behavior, which in turn affect the copper smelting efficiency and effect. In the present work, the predicted hydrodynamics and mixing phenomenon would be verified by the water model experiment, and then the nozzle layout and bottom blowing dynamics would be investigated and optimized.

### 2.1. Model Validation

**Figure 2:**
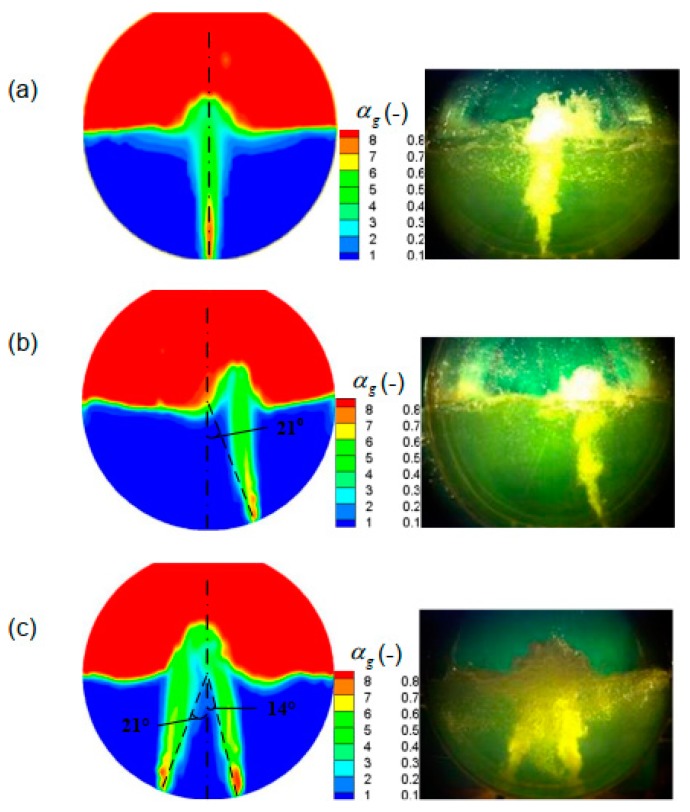
Comparison of the predicted local gas volume fraction with experimental photos in the bath with different nozzle arrangement. The angles of the nozzle arrangement are (**a**) β= 0°, (**b**) β= 21°, and (**c**) β_A_ = 14°, and β_B_ = −21°, respectively.

**Figure 3:**
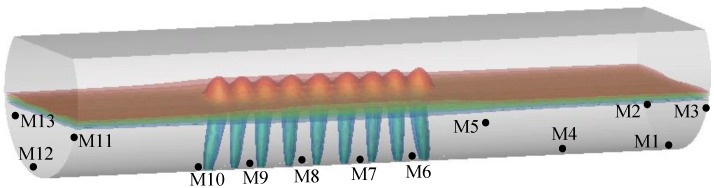
Arrangement of tracer monitoring points (M1 to M13) in a bath.

Figure 2 shows a comparison of experimental photos and simulated predictions of gas-liquid two-phase distribution in molten pool. In this figure, the total gas flow rate is 4.7 m^3^/h and the liquid level is 342 mm. β is the angle between the nozzle normal arrangement and the bottom vertical centerline of the furnace. It can be seen from Figure 3 that for the different experimental conditions, the predicted gas–liquid two-phase region in the molten pool are in good agreement with the experimental photographs, and the present model performs well in predicting bubbly plume flow in bottom blown cooper smelt furnace. 

The mixing process of homogenizing the concentration is directly related to the efficiency of metallurgical reactions in the molten pool, and the mixing time can be obtained when the local tracer concentrations of all measuring points reach within 5% deviation of the homogeneous value. Some studies have shown that mixing time is directly affected by the position of the measuring point which need be located in the dead zone [13,14,15]. Due to the large axial dimension of the furnace itself, in order to more accurately reflect the mixing process in the furnace, 13 tracer monitoring points (M1–M13) are selected in the present work, as shown in Figure 3.

**Figure 4:**
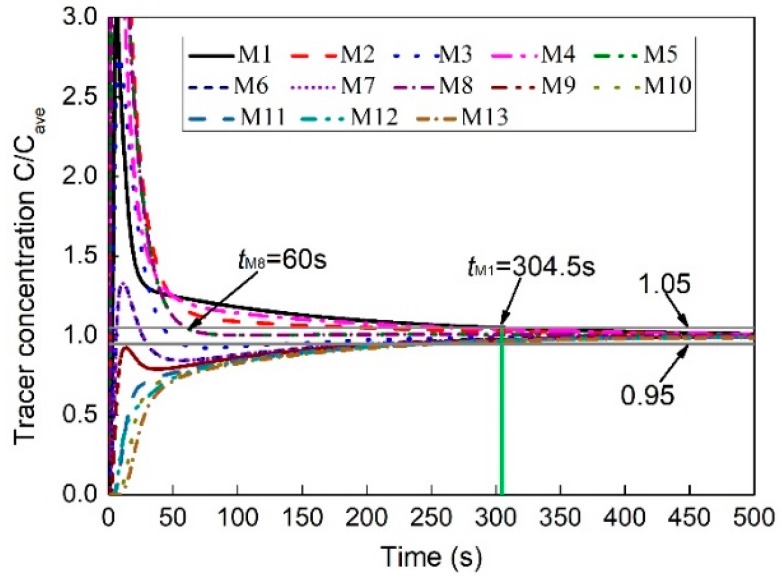
Predicted variation of tracer mass concentration of the thirteen monitoring points (M1–M13) with time after the tracer added to the bath.

Figure 4 gives the predicted tracer mass concentration of the thirteen measuring point (M1 to M13) in the furnace respectively, and the *C*/*C_ave_* is a dimensionless mass concentration which refers to the ratio of the local value to the homogeneous. From this figure, it can be found that the times when the *C*/*C_ave_* of each measuring point finally reaches the range of 0.95–1.05 is different, and the monitoring points M8 and M1 are the first and last points reaching the range of 0.95–1.05, responsibility. In this figure, the *t*_M8_ and *t*_M1_ are 60 s and 304.5 s, respectively. Therefore, the mixing time of the entire molten pool is 304.5 s, and it is necessary to select multiple monitoring points for accurate mixing efficiency in the molten pool. 

**Figure 5:**
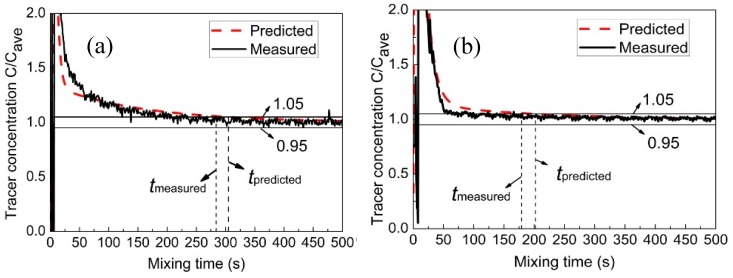

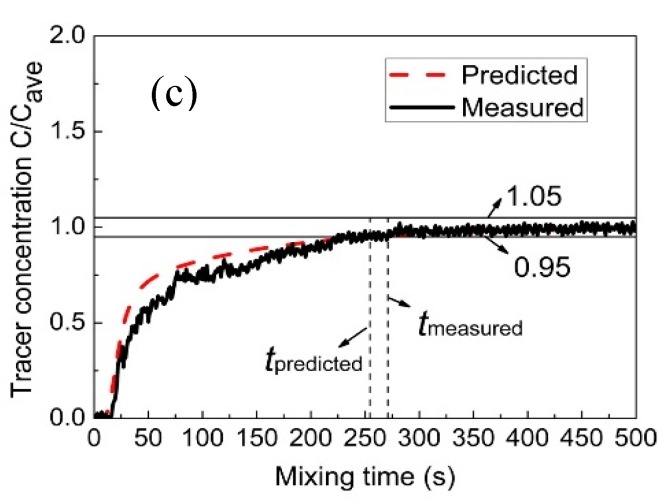
Measured and predicted tracer dimensionless concentration of measuring points (**a**) M1, (**b**) M6, and (**c**) M13 with time in bath.

Figure 5 illustrates the measured and predicted variation of $C/C_{ave}$ over time in the furnace. It should be noted that it is difficult to simultaneously measure tracer concentration at many points because of the limitation of physical experiments. In order to verify the predicted mixing phenomenon, only the three measuring points (M1, M6, and M13), which are located at the center and ends of the furnace respectively, as shown in Figure 4, are selected to measure the mixing time. It can be found from this figure that the predicted mixing process is in good agreement with the measured data at the same measuring points. In the end regions of the molten pool, such as monitoring points M1 and M13, the mixing efficiency of the components is low, especially in the right end region (M1), where it is furthest from the gas-stirred zone. In the middle of the molten pool, such as monitoring point M6, the mixing efficiency is higher. 

### 2.2. Effect of the Nozzle Arrangement

**Figure 6:**
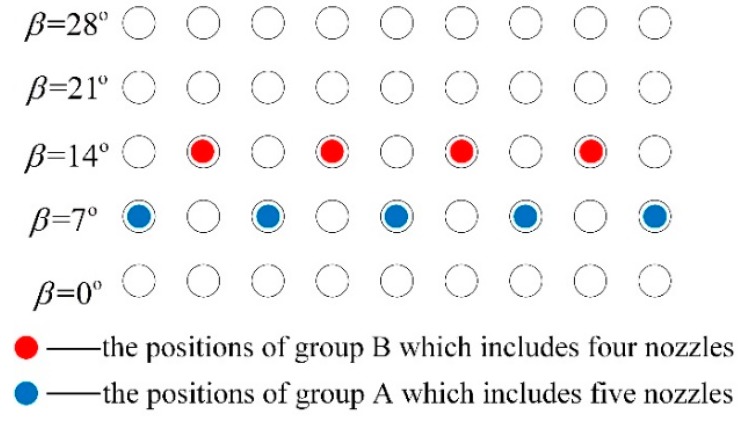
Schematic of the nozzle arrangement of group A and group B at the bottom of the bath.

The nozzle arrangement has a significance effect on the gas–liquid two-phase flow and mixing behavior. In the present work, nine nozzles were adopted and divided into two groups, A and B, and the arrangement angle between the nozzles and the center of the furnace was investigated from 0° to 28°, as shown in Figure 6. Different nozzle placement schemes were distinguished by changing the arrangement angle of the group A and group B, i.e., β_A_ and β_B_.

**Figure 7:**
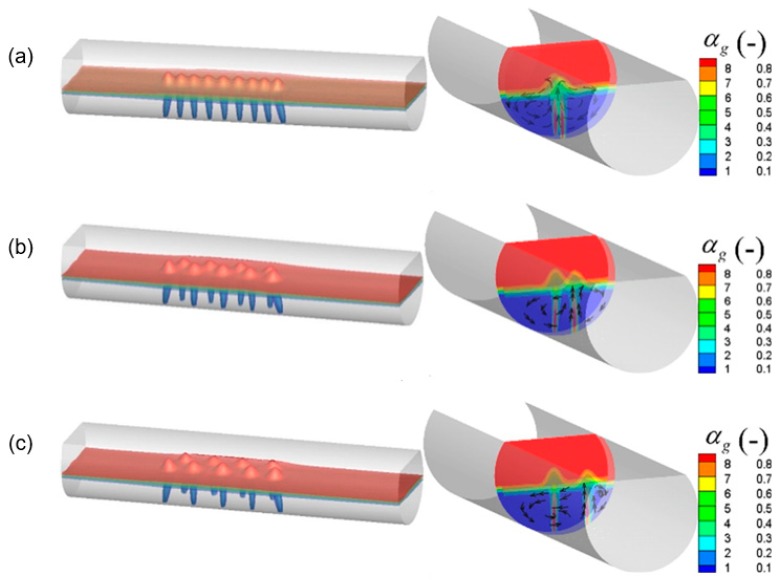
Predicted typical local gas volume fraction in the bath with different nozzle arrangements, where the β_A_ is 0°, and the β_B_ is (**a**) 0°, (**b**) 14°, and (**c**) 28°, respectively.

**Figure 8:**
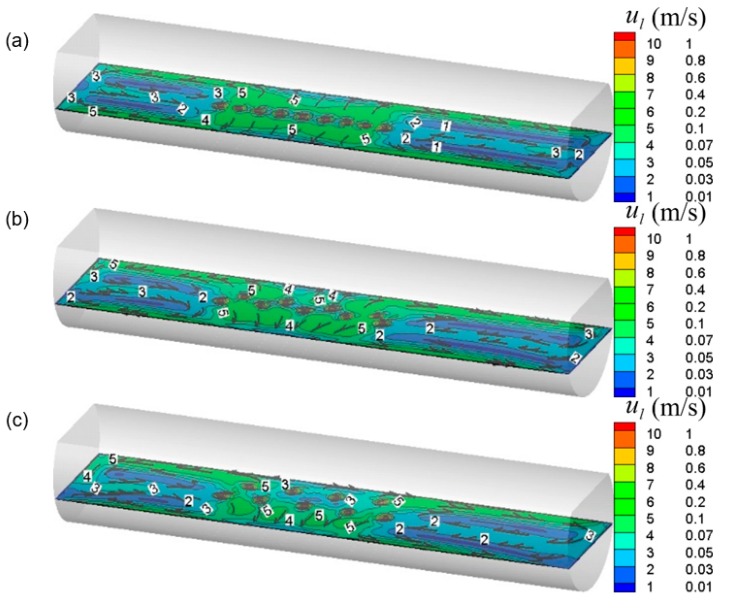
Predicted typical local liquid flow filed in the bath with different nozzle arrangements, where the β_A_ is 0°, and the β_B_ is (**a**) 0°, (**b**) 14°, and (**c**) 28°, respectively.

Figure 7 and Figure 8 show the predicted typical gas–liquid two-phase flow under different nozzle arrangement in the overall molten pool. In this figure, the nine nozzles are adopted, of which five nozzles are arranged in group A and four nozzles are arranged in group B. The nozzle placement angle of group A is 0°, and the nozzle placement angles of group B are 0°, 14°, and 28°, respectively. The total gas flow rate is 9.4 Nm^3^/h. It can be seen from Figure 7 that when both groups A and B are arranged at 0°, the bubbly plume flow in the center of the molten pool drives the metal melt to form a symmetrical circulation motion. As the β_B_ increases, the bubble stream is separated from each other and the bubble stream gradually moves toward the side wall, where the circulation area between the stream and the wall surface also decreases. 

From Figure 8, in the gas injection zone, the intense liquid flow is driven by bubble agitation from the center to the side wall of the molten pool. At the axial ends of the molten pool, the liquid flow rate is weak, and two symmetrical circulating motions are formed along the axial direction. Furthermore, it is also noted that as the β_B_ gradually increases, the bubble flow gradually approaches the wall surface, and accordingly the symmetry of the individual circulation flow is gradually broken due to displacement of the jet, which would facilitate the transport of components throughout the molten pool. But when the β_B_ is too large, the low velocity zone is formed between the bubble streams because the bubble floating distance become smaller, as shown in Figure 8c. 

For a gas-stirred reactor, the longer the bubbles stay in the molten pool, the larger the gas total volume, and the higher the gas–liquid reaction efficiency in the molten pool. In present system, the gas total volume, *θ*, of the furnace is given by integrating the local gas volume fraction, *α_g_* and cell volume, *Vcell*, throughout the molten pool:

(1) $$\theta =\int \alpha_{g}dV_{cell}.$$

**Figure 9:**
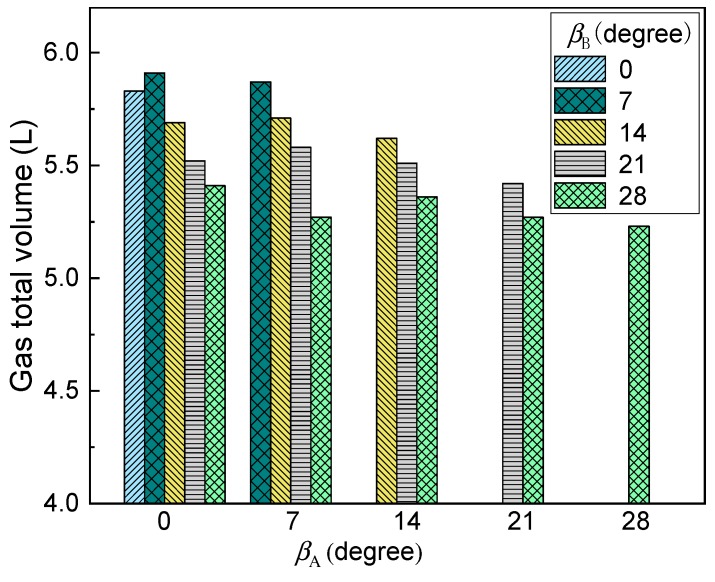
Effect of different nozzle arrangement on the predicted gas total volume in the bath.

Figure 9 gives the predicted gas total volume in the bath under different nozzle placement angles. In this figure, the nine nozzles are adopted, of which five nozzles are arranged in group A and four nozzles are arranged in group B. It can be found from this figure that with the increasing nozzle placement angles, the gas total volume in the bath gradually decreases. It is worth noting that when all nozzles are placed at the center of the bottom of the furnace, i.e., β_A_ = β_B_ = 0, the gas volume is not the highest. When there is a certain deviation between the two nozzle arrangements, i.e., β_B_ is 7°, the total volume of gas in the molten pool is the largest. This is mainly because when β_B_ is 0°, the distance between the two bubble streams is small, and the flows in the upper part of the molten pool overlap each other, as shown in Figure 8a, which would reduce the bubble utilization. With the β_B_ increase to 7°, the bubble dispersibility becomes better, and the bubble residence time becomes larger. In addition, it can be also seen from the figure that when the nozzle arrangement exceeds 14°, the gas volume is small, which is disadvantageous for the gas residence time.

**Figure 10:**
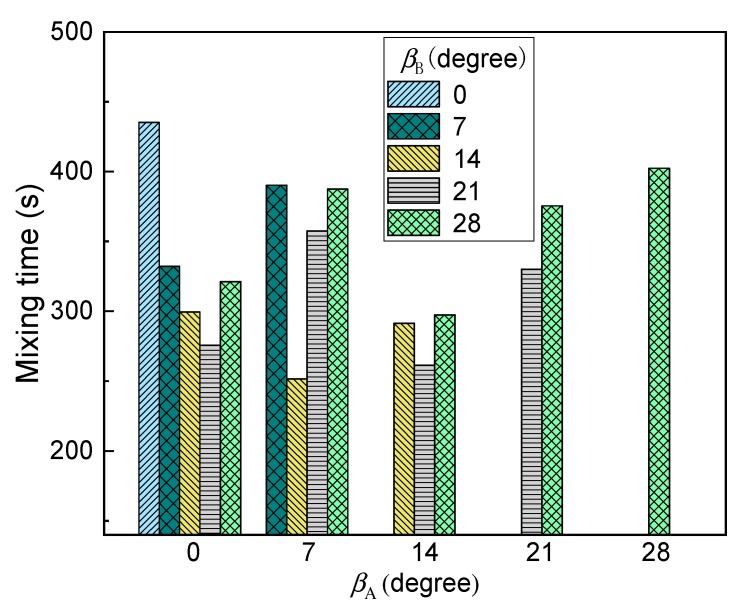
Effect of different nozzle arrangements on the predicted mixing time in the bath.

Figure 10 shows the effect of different nozzle arrangements on the predicted mixing time in the molten pool. It can be seen from the figure that with an increase in nozzle placement angles, the mixing time decreases first, and then increases. When the nozzles of both groups A and B are located in the center of the furnace, the mixing time in the molten pool is the longest, i.e., *t*_mix_ = 435.2 s and when β_A_ and β_B_ are 7° and 14°, respectively, the mixing time is the shortest, i.e., *t*_mix_ = 251.5 s. This is mainly because when both β_A_ and β_B_ are 0°, the mutually symmetric and independent circulation regions are formed, as shown in Figure 9a, and it is difficult for tracer transport between the independent circulation flows. As the nozzle arrangement angle gradually increases, the symmetry of the individual circulation flow is gradually broken due to displacement of the jet, and the mixing time in the molten pool gradually decreases. However, when the nozzle arrangement exceeds 21°, the nozzle position of group B is closer to the liquid surface, and the bubble floating distance shortened. Therefore, the mixing efficiency of the molten pool induced by gas-stirred is also gradually reduced. Furthermore, it should be noted that in the current actual production process, β_A_ and β_B_ are 7° and 21°, respectively, and the predicted *t*_mix_ is 357.5 s, therefore, the existing layout is unreasonable, and the nozzle angles of the group A and B arrangement at 7° and 14° are recommended.

### 2.3. Effect of the Nozzle Number

For the bottom blowing copper smelting furnace, it is of great significance to select a reasonable nozzle number to improve the smelting efficiency and economic benefits. In the present work, the effects of 9, 13, and 18 nozzles on the gas–liquid flow and mixing time are studied, and these nozzles are divided into two groups, A and B. The total gas flow rate of 9.4 Nm^3^/h is adopted. According to the above research, the β_A_ of 7° and β_B_ of 14° are adopted, and for nine nozzles, five nozzles are arranged in group A and four nozzles are arranged in group B. For 13 nozzles, nine nozzles are arranged in group A and four nozzles are arranged in group B. For 18 nozzles, nine nozzles are arranged in group A and group B, respectively.

**Figure 11:**
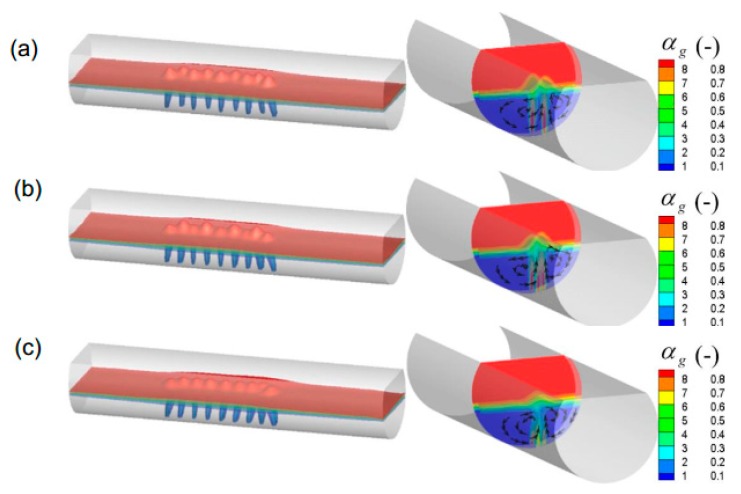
Predicted local gas volume fraction in the bath with different numbers of nozzles, and the nozzle numbers are (**a**) 9, (**b**) 13, and (**c**) 18, respectively.

**Figure 12:**
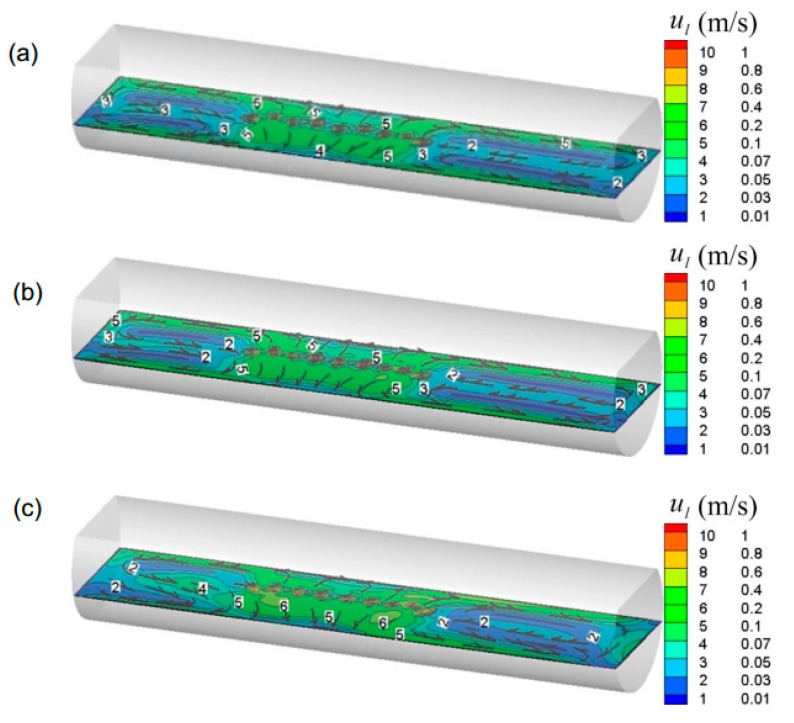
Predicted local liquid flow filed in the bath with different numbers of nozzles, which are (**a**) 9, (**b**) 13, and (**c**) 18, respectively.

Figure 11 and Figure 12 show the effect of nozzle number on the predicted gas–liquid two-phase flow in the molten pool. It can be seen from these figures that with the increase of nozzle number from nine to 18, the bubble dispersion becomes better, and the standing wave height of the bubble column on the liquid surface gradually decreases. In Figure 12, with the nozzle number increasing from nine to 13, the flow tendency in the furnace does not change much. However, when the nozzle number is increased to 18, the liquid velocity is slightly enhanced in the gas injection zone due to better bubble dispersion, but the low velocity zone begins to become larger at the right end of the furnace.

**Figure 13:**
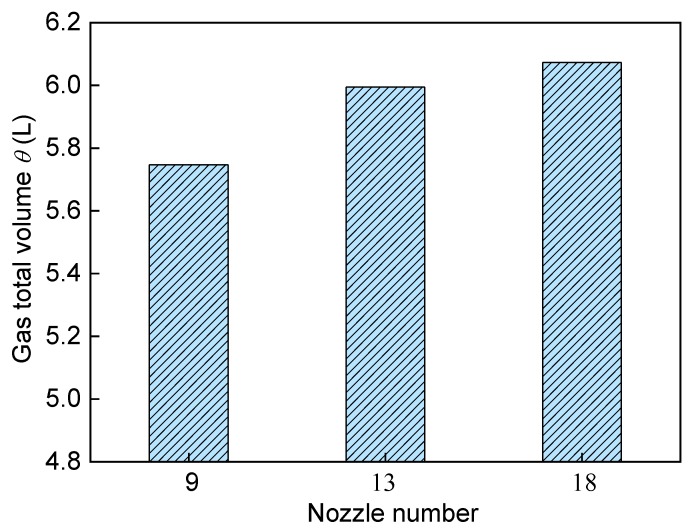
Effect of different nozzle numbers on the predicted gas total volume in the bath.

Figure 13 show the effect of different nozzle numbers on the predicted gas total volume in the bath, with the increase of nozzle number, the gas total volume in the molten pool gradually increases because the bubble dispersion becomes better, and when the nozzle number exceeds 13, the gas total volume changes little.

**Figure 14:**
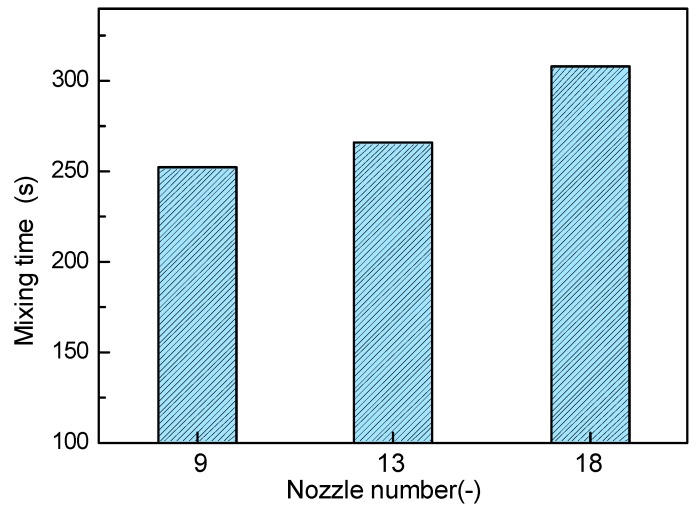
Effect of different nozzle number on the predicted and measured mixing time in the bath.

Figure 14 shows the effect of nozzle number on the mixing time in the molten pool. It can be seen from the figure that as the number of nozzles increases from nine to 13, the mixing time increases slightly, but when the number of nozzles is further increased to 18, the mixing efficiency begins to decrease significantly. This is because with the increasing of nozzle number, the bubble dispersibility is improved, and the flow velocity in the central portion of the molten pool is relatively uniform, but in the end region, the inhomogeneity of liquid flow is enhanced, especially for 18 nozzles, as shown in Figure 13.

### 2.4. Effect of the Gas Flow Rate

**Figure 15:**
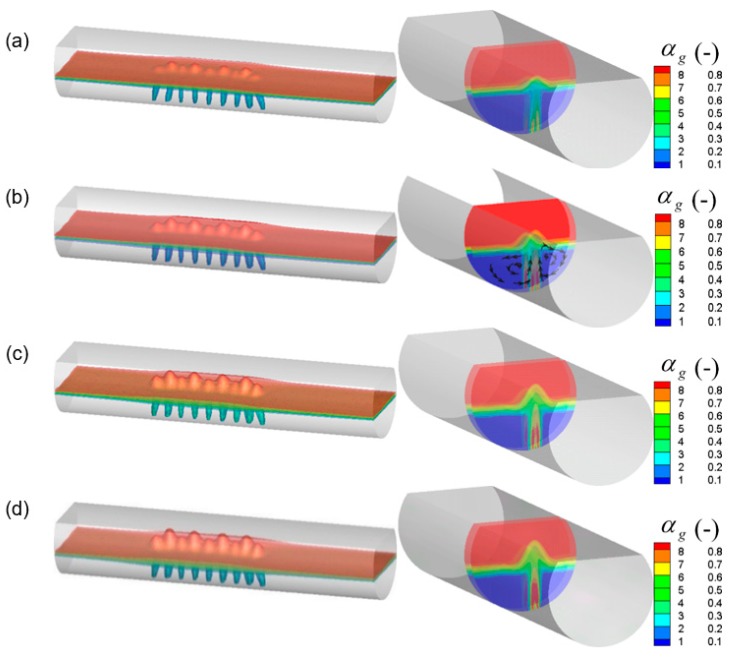
Predicted local gas volume fraction in the bath with different gas flow rates, which are (**a**) 4.7 m^3^/h, (**b**) 9.4 m^3^/h, (**c**) 14.1 m^3^/h, and (**d**) 18.8 m^3^/h, respectively.

**Figure 16:**
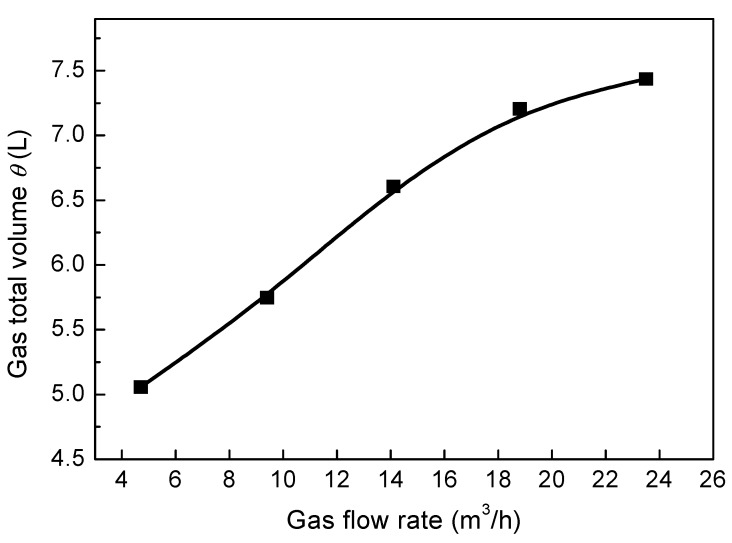
Effect of different gas flow rates on the predicted gas total volume, *θ*, in the bath.

The gas flow rate is an important parameter that directly relates to the gas–liquid reaction efficiency and component mixing efficiency of bottom blown copper smelting furnaces. Figure 15 and Figure 16 show the effect of different gas flow rates on the predicted gas–liquid flow and gas total volume in the furnace. In these figures, the 13 nozzles are adopted at the bottom of the furnace, where nine nozzles are arranged in group A and four nozzles are arranged in group B. β_A_ and β_B_ are 7° and 1°, respectively. It can be seen from the figure that with the gas flow rate increasing, the gas volume fraction in the bubble plume zone gradually increases, and the standing wave height of the bubble column also gradually increases. When the gas flow rate exceeds 18.8 m^3^/h, the two bubble streams at 7° and 14° converge into one large stream, as shown in Figure 15d. Furthermore, it can also be found that with the increase of gas flow rate from 4.7 m^3^/h to 18.8 m^3^/h, the gas total volume in the molten pool increases from 5.06 L to 7.21 L, and when the gas flow rate exceeds 18.8 m^3^/h, the growth rate of gas total volume becomes smaller because the two streams overlap each other, as shown in Figure 15d.

**Figure 17:**
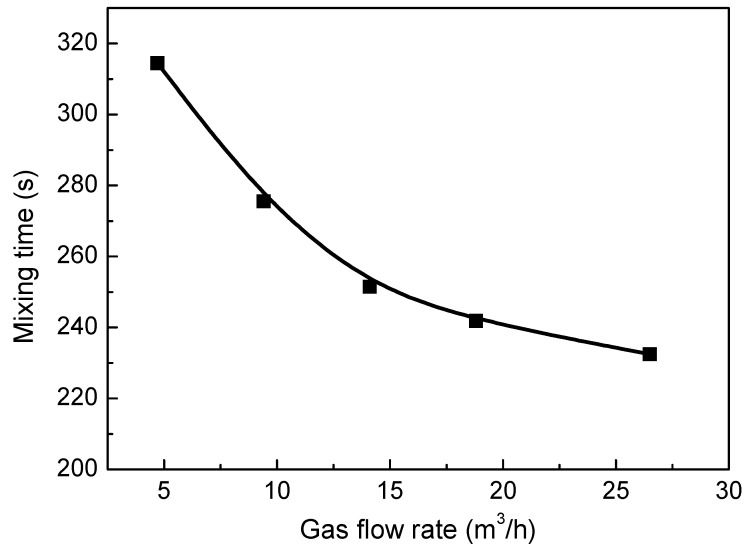
The effect of different gas flow rate on the predicted mixing time in the bath.

Figure 17 shows the effect of gas flow rates on the predicted mixing time in the molten pool. It can be seen from this figure that at lower blowing gas rates, the mixing efficiency of the molten pool is lower, and the mixing time is 314.5 s for the gas flow rate of 4.7 m^3^/h. With the gas flow rate increasing from 4.7 m^3^/h to 14.1 m^3^/h, the mixing time is rapidly reduced from 314.5 to 251.5, but when the gas flow rate exceeds 14.1 m^3^/h, the mixing time changes little. This is because at the larger gas flow rate, the bubble streams at 7° and 14° converge into one large stream, and the bubble dispersion is weakened. As well, in the axial end regions of the molten pool, the liquid flow is less affected by the bubble motion.

## 3. Materials and Methods

### 3.1. Water Model 

In order to verify the simulation results and study the overall flow and mixing efficiency in the furnace, a scaled-down 1:9.3 water model was constructed, and the related water model geometry parameters and material properties are given in Table 1. In the water model experiment, the range of gas flow rate for the present water model is calculated based on the Froude Number [10,11,12]. The bubble behavior and distribution in the water model of bottom blown oxygen copper furnace was captured by a high speed camera (1000 frame/s). In order to reflect the overall flow mixing effect in the furnace, 100 mL of saturated KCl solution was added each time, and then the mixing state of the KCl can be obtained through the conductivity metering, which can record the real-time change data of local conductivity of the aqueous solution.

### 3.2. Governing Equations for the Mathematical Model 

The governing equations of the Euler–Euler model are listed in Table 2. In the present system, the effect of top slag on fluid flow is neglected, and only gas–liquid two phase flow is considered. In Table 2, *ρ_i_*, *α_i_*, and *u_i_* are the density, volume fraction, and velocity vector of the liquid phase (*i* = l) and gas phase (*i* = g), respectively. The multiple interaction forces, *M_l_*, are considered, namely drag force, *F_D_*, lift force, *F_L_*, virtue mass force, *F_VD_*, and turbulent dispersion force, *F_TD_* [16,17]. C_Dvis_, C_Ddis,_ and C_Dcap_ represent the drag force coefficient calculated in the viscous regime, distorted bubbles regime, and capped bubbles regime, respectively. *E_o_’* is Eötvös number, and *σ* is surface tension. The *κ-ε* turbulence model was adopted to describe the gas–liquid turbulent behavior in the bottom blown copper smelting furnace. Furthermore, in order to describe the mixing process in a bottom blown copper smelting furnace, the tracer transport equation is solved to predict the local distribution of tracer concentrations, and then the mixing time can be obtained once the tracer concentrations of all monitoring points are within 5% deviation of the homogeneous value [10,13,14,15].

### 3.3. Boundary Conditions and Numerical Scheme

**Figure 18:**
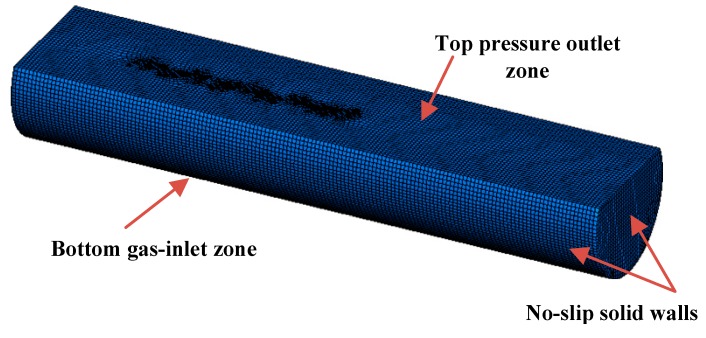
Geometry and mesh of bottom blown copper smelter water model.

Figure 18 shows the geometry and mesh of a bottom blown copper smelter water model, and a total of about 302,000 hexahedral meshes were created to develop the flow computational domain. In the present model, the upper part near the top feed port of the furnace is mainly the gas phase zone, which is ignored in order to improve the calculation efficiency. Furthermore, because of the large velocity gradient of the gas–liquid flow near the nozzles at the bottom of the furnace, the meshes are encrypted in this area to ensure the accuracy and stability of the calculation results. For the boundary conditions, all the nozzles at the bottom of furnace are set as gas velocity-inlet, and the initial gas velocity is calculated based on the gas flow rate. The top surface of the furnace is set as the pressure-outlet, where the pressure is set to ambient atmospheric pressure. The bottom and sides of the furnace are treated as solid walls. The mixing process can be evaluated by introducing a small amount of tracer into the furnace at steady-state flow regime (t = t_0_) and then monitoring its dispersion at sampling positions with time (t–t_0_). In the present model, the flow regime with blowing time t_0_ of 200 s was used to calculate the mixing process in bottom blown copper smelting furnace. All control equations in this paper are calculated using CFD (Computational Fluid Dynamics) commercial software Fluent 18.0, and the convergence is marked by a dimensionless residual of less than 1.0 × 10^−4^ for each variable. The time step is taken is 0.002 s, and the entire calculation process needs to calculate 30,000 time steps.

## 4. Conclusions

A mathematical model was developed to describe gas and liquid two-phase flow and mixing behavior in a bottom blown oxygen copper smelting furnace, and the model validation is carried out through the water model experiment. The effects of different nozzle locations, nozzle numbers, and gas flow rates on the gas-liquid flow, gas total volume, and mixing time in the furnace were investigated. The results indicate that:

(1) When the nozzles are located near the center of the bath bottom, the gas total volume *θ* is larger, but the mixing efficiency is very low. With the increase of nozzle arrangement angle, the mixing time gradually decreases. However, when the nozzle angle arrangement exceeds 21°, the distance for bubbles traveling from the nozzle to the liquid surface is shortened, which will reduce the gas total volume. For the present system, the nozzle angles of the group A and B arrangement at 7° and 14° are recommended.
(2) With the increasing of number of nozzles from nine to 13, the gas total volume in the furnace increases due to better bubble dispersion, and the mixing efficiency does not change greatly. However, when the number of nozzles is further increased to 18, the gas total volume changes little, and the mixing efficiency begins to decrease significantly. Therefore, the number of nozzles recommended is 13.
(3) With the gas flow rate increasing, the gas total volume fraction in the furnace increases, and when the gas flow rate exceeds 18.8 m^3^/h, the growth rate of gas total volume becomes smaller because the two streams overlap each other. With the gas flow rate increases from 4.7 m^3^/h to 14.1 m^3^/h, the mixing time is rapidly reduced from 314.5 s to 251.5 s, however, when the gas flow rate exceeds 14.1 m^3^/h, the mixing time change little. Therefore, it is recommended that the maximum gas flow rate does not exceed 18 m^3^/h.

## Figures and Tables

**Table 1:** Related model geometry parameters and material properties.

	Industrial Prototype	Water Model
Diameter of furnace (mm)	3420	367.7
Length of furnace (mm)	15,000	1612.9
Liquid level (mm)	1710	183.9
Density of liquid (kg/m^3^)	4600	1000
Gas density (kg/m^3^)	1.37	1.25
Gas flow rate (m^3^/h)	4000–12,000	6.74–20.24

**Table 2:** Governing equations of the mathematical model.

Models	Remarks	Equations
Continuity conservation	Gas phase: i = g Liquid phase: i = l	$\frac{\partial \left(\alpha_{i}\rho_{i}\right)}{\partial t}+\nabla \cdot \left(\alpha_{i}\rho_{i}u_{i}\right)=0$
Momentum conservation		$\frac{\partial \left(\alpha_{i}\rho_{i}u_{i}\right)}{\partial t}+\nabla \cdot \left(\alpha_{i}\rho_{i}u_{i}u_{i}\right)=-\alpha_{i}\nabla p+\nabla \cdot \left(\alpha_{i}\mu_{eff}\left(\nabla {\bar{u}}_{i}+{\left(\nabla {\bar{u}}_{i}\right)}^{T}\right)\right)+\alpha_{i}\rho_{i}g+M_{i}$ $M_{l}=-M_{g}=F_{D}+F_{VM}+F_{L}+F_{TD}+F_{W}$
Interphase forces	Drag force	$F_{D}=\frac{3\alpha_{g}\alpha_{l}\rho_{l}C_{D}}{4d_{g}}\left|u_{g}-u_{l}\right|\left(u_{g}-u_{l}\right)$ {CDvis=24/Re(1+0.1Re0.75)if CDdis<CDvis,CD=CDvisCDdis=2/3((gρl)0.5dgσ0.5)(1+17.67(1−αg)1.28618.67(1−αg)1.5)2if CDvis<CDdis<CDcap,CD=CDvisCDcap=3/8(1−αg)2if CDdis>CDcap,CD=CDcap
	Virtue mass force	${\vec{F}}_{VM}=\alpha_{g}\rho_{l}C_{VM}\frac{D\left(u_{g}-u_{l}\right)}{Dt}$, $C_{VM}=0.5$
	Lift force	${\vec{F}}_{L}={-C}_{l}\rho_{l}\alpha_{g}\left(u_{l}-u_{g}\right)\times \left(\nabla \times u_{l}\right)$ Cl={min[0.288tanh(0.121Re),f(Eo′)]Eo′≤4f(Eo′)4<Eo′≤10−0.2710<Eo′ $f\left(Eo^{\prime }\right)=0.00105Eo^{\prime 3}-0.0159Eo^{\prime 2}-0.0204Eo^{\prime }+0.474$ $Eo^{\prime }=g\left(\rho_{l}-\rho_{g}\right)d_{g}^{2}/\sigma$
	Turbulent dispersion force	${\vec{F}}_{TD}^{g-p}=-\frac{3\alpha_{g}\alpha_{l}\rho_{l}C_{D}}{4d_{g}}{\vec{u}}_{drift}$ ${\vec{u}}_{drift}=\frac{D_{gl}^{t}}{\omega_{gl}}\left(\frac{1}{\alpha_{l}}\nabla \alpha_{l}-\frac{1}{\alpha_{g}}\nabla \alpha_{g}\right)$
	Wall lubrication force	${\vec{F}}_{W}={-C}_{l}\rho_{l}\alpha_{g}\left(u_{l}-u_{g}\right)\times \left(\nabla \times u_{l}\right)$
Turbulent model	k equation	$\frac{\partial }{\partial t}\left(\alpha_{l}\rho_{l}k\right)+\nabla \cdot \left(\alpha_{l}\rho_{l}{\vec{u}}_{l}k\right)=\nabla \cdot \left(\alpha_{l}\frac{\mu_{t,l}}{\sigma_{k}}\nabla k\right)+\alpha_{l}G_{k,l}+\alpha_{l}G_{b}-\alpha_{l}\rho_{l}\varepsilon$
	ε equation	$\frac{\partial }{\partial t}\left(\alpha_{l}\rho_{l}\varepsilon \right)+\nabla \cdot \left(\alpha_{l}\rho_{l}{\vec{u}}_{l}\varepsilon \right)=\nabla \cdot \left(\alpha_{l}\frac{\mu_{t,l}}{\sigma_{\varepsilon }}\nabla \varepsilon \right)+\alpha_{l}\frac{\varepsilon }{k}\left(C_{1\varepsilon }(G_{k}+G_{b})-C_{2\varepsilon }\rho_{l}\varepsilon \right)$
	Liquid velocity gradients induced turbulence	$G_{k,l}=\mu_{t}\left(\nabla {\vec{u}}_{l}+{\left(\nabla {\vec{u}}_{l}\right)}^{T}\right):\nabla {\vec{u}}_{l}$
	Bubble induced turbulence [13]	$G_{b}=C_{b}\frac{\mu_{t}}{\mu_{eff}}\left(\rho_{l}-\rho_{g}\right)gV_{B}{\vec{u}}_{rel}$
Tracer Transport Equation	C is the local trace mass fraction	$\frac{\partial }{\partial t}\left(\rho C\right)+\nabla \cdot \left(\rho u_{l}C\right)=\nabla \cdot \left(\frac{\mu_{t}}{Sc_{t}}\left(\nabla C\right)\right)$
